# Dr. Lambe's Additonal Reports on the Effects of a Peculiar Regimen, &c. &c.

**Published:** 1816-12

**Authors:** 


### Dr. Lambe's *Additonal Reports on the Effects of a peculiar Regimen, &c. &c.*

( Continued from page 385. )

The remaining portion of this volume is dedicated to a detail of cases, in elucidation of the principles advanced in our former analysis. The first is, that of our author himself; and under the title "*Podagra Atonica,*" he gives a morbid history of his own life, of which we shall endeavour to exhibit a sketch, as we consider it very interesting.

*Aug.* 9*th,* 1813. *Ætat.* 49. First eighteen years of life free from disease. After a severe wound in the forehead, at 8 years of age, he could not bear a strong light; he was of a lax fibre; feeble pulse; thin, pale, delicate. At 18, an eruption of pimples on the face, neck, shoulders, and breast, which continued twenty years, accompanied by dyspeptic symptoms. At 28, sudden and transient lameneses. Till 32, he had only occasional dyspepsia; sick head-aches; impatience of light; lumbago; rheumatism. At 32, confusion about the head; giddiness after reading; inaptitude for study; heat in the head at night. Irregularity of bowels, and increase of dyspeptic symptoms now took place. Aperient medicines became constantly necessary; hypochondriasis. In January 99, severe enteritis, after which pain continued in the right epigastrium for a twelvemonth, with abdominal tenderness. For two years, pretty good health, except dyspepsia, constipation, and flatulence, with occasional hypochondriacal horrors.

In 1803, the uneasiness of stomach had increased to a great height; and Dr. L resolved all at once to substitute distilled for common water, by which the morbid feelings of the stomach were much mitigated. In nine months, the sick head aches left him, and have not since returned. An annual periodical attack of face-ache, which he had experienced for several years in the months of October, also disappeared. In 1804, another attack of enteritis, more easily subdued. A whole host of anomalous morbid symptoms also vanished under the use of the distilled water. But this immunity was only temporary; they all returned with some new ones: and towards the close of 1805, he was a victim to infirmities; he therefore now resolved to relinquish animal food, from which he has wholly refrained up to the present period.

"He never found the smallest real ill consequence from this change. He neither sunk in strength, flesh, nor spirits. He was at all times of a very thin and spare habit; and so he continued to be; but, upon the whole, he has rather gained than lost flesh. He has experienced neither indigestion, nor flatulence, even from the sort of vegetables, which are commonly experienced to be the most oppressive and windy; as beans, peas, peas-soup, &c. Nor has the stomach suffered from any vegetable matter, though unchanged by culinary art, or uncorrected by condiments. These results, so opposite to common experience, and even to his own, in the former part of life, can be accounted for only by considering the changes introduced into the state of the digesting organs by the previous use of the purified water. The only unpleasant consequence of the change was a sense of emptiness of the stomach, which continued many months. In about a year, however, he became fully reconciled to the new habit; and felt as well satisfied with his vegetable meal as he had been formerly with his dinner of flesh." p. 291.

Although the good effects of this change have been progressive, they have been slowly so; and Dr. L. thinks, from the long and obstinate head-aches experienced, that organic lesion of the brain had commenced, and was going on "towards an apoplectic or paralytic attack," had it not been arrested by the plans detailed above. He thinks, and probably with great justice, that bleeding, blistering, and the various depletory methods resorted to in these cases, would have only given temporary relief, and that the vegetable regime alone could have effectually checked the advance of the disease.

"But although these pains still recur in a trifling degree, the relief given to the brain in general, has been decided and most essential. It has appeared in an increased sensibility of all the organs, particularly of the senses, the touch, the taste, and the sight; in greater muscular activity; in greater freedom and strength of respiration; greater freedom of all the secretions; and an increased intellectual power. It has been extended to the night as much as to the day. The sleep is more tranquil, less disturbed by dreams, and more refreshing. Less sleep upon the whole appears to be required. But the loss of quantity is more than compensated by its being sound and uninterrupted." p. 300.

It is somewhat curious, that shortly after Dr. Lambe had embraced the vegetable system of diet, gouty affections of the lower extremities manifested themselves, and continued for several years to increase as the other constitutional or local sypmtoms of disease declined. From this fact our author concludes, that "disease, though seated in different organs, may be the same in kind, and that it is the property of this regimen, and in particular, of the vegetable diet, to transfer the diseased lesion from the viscera to the exterior parts of the body." We firmly believe this to be true; and the circumstance accounts for the charge against vegetable diet of causing cutaneous eruptions—"from poorness of blood;" a charge which, instead of being a reproach, is a proof of salubrity.

*Phthisis.*—In every case of *confirmed* pulmonary consumption which Dr. L. has seen, death was the result. We fear this is the general experience of the profession! In some, the benefit for a time, was so striking as to raise hopes of recovery, but these hopes were fallacious. Dr. L. is still of opinion, that phthisis is not a local disease of the lungs, but a constitutional disease of the whole body. He justly observes, that incipient phthisis is with difficulty recognised sometimes, and that patients are often on the verge of the grave before they think themselves seriously ill. It is, therefore, difficult to say how far regimen possesses a preventive power over the disease. Under a vegetable diet and pure water, however, the chest takes a more perfect and expanded form; a strong argument in its favour, since a contracted thorax is the strongest of all external signs of a consumptive tendency. The following case occurring under Dr. L's own roof, he considers as affording satisfactory evidence of the power of the regime in question, in arresting the progress of consumption.

M. W. ætat. 33, a female, had lived with Dr. L. thirteen years. At 20, she had no particular complaint, but was subject to convulsive and hysterical affections; occasionally to cough. In 1807, she evinced strong signs of failing health; the colour forsook her cheeks; the appetite failed; the muscular strength was impaired. Dr. L. advised restriction in drink to pure water alone; in food, to vegetables. She would only conform to this rule every second day for six weeks. In November 1808, she was found to have dyspnœa; inability to expand the chest; or lie but on one particular side: anhelation on ascending the stairs; pain in the side; occasional cough. These symptoms, connected with her generally impaired state of health, indicated, with much certainty, approaching phthisis. The vegetable and aqueous regime was now insisted on. During 1809, the general health somewhat improved; as did the appetite; but the symptoms before stated remained stationary. In the latter part of 1810, the relief became decisive. She could draw breath fully and freely; could lie on either side. By the latter end of 1812, having rigidly adhered to the antediluvian system all this time, she was quite recovered in health and looks, and left her service abruptly in a state of pregnancy.

Under the head of *Asthma Convulsivum,* Dr. L. has introduced a sketch of the medical history of Mr. Newton, a gentleman well known by his publication entitled "The Return to Nature," an abstract of which history we shall present to readers. Mr. N. is the father of a beautiful family of children, bred up, with regard to diet, on the principles under consideration. He has been subject to asthmatic attacks since the age of seven years. These attacks usually continued from one week to three, during whichhe could not lie down in his bed. The intervals seldom exceeded three months, and even in these there was a constant uneasiness in the breast upon inspiration. In 1805, at which time he was a martyr to the disease, he adopted Dr. L's advice of using distilled water; the change was immediately beneficial; the general health improved; and he experienced but two attacks of short duration, in two years and a half.

Towards the close of 1807, at Dr. L's suggestion, he began to wean himself from animal food. During the next three years he had very little asthma; but his general health was in a very critical situation. The pulse was commonly very rapid, even to 120, with quickness of respiration, and copious mucous defluxions. Nevertheless his health gradually and progressively amended under the vegeto-aqueous regimen. In May, the lungs became loaded with phlegm, with heaviness of the head, and excessive itching about the eyes. Anhelation on ascending the stairs. These were succeeded by severe paroxysms of asthma, swelled ankles, great stricture on the chest, &c. An expectoration coming on, free and copious, however, he was restored to health in about two months. Mr. N. persevered in his habits with unabated zeal, and has received the due reward of his confidence and perseverance.

He enjoyed an immunity from asthma till the same period in next June, when he experienced another relapse, which has regularly recurred every year since. He adheres, however, to his regimen with greater strictness than ever. He often makes his dinner on a little fruit, dried raisins, bread, and three or four potatoes; and upon this strict course of abstinence has found no defect of strength or nutrition. On the contrary, the symptoms with which he has been occasionally affected, have been accompanied with marks of plenitude and oppression. 350.

Dr. Lambe thinks, perhaps with justice, that Mr. Newton's life has been prolonged, in consequence of this regular establishment of periodical asthma by the vegeto-aqueous regimen.

"Whatever (says he) is a person's habitual disease, is to that person, relatively, a state of health; and such disease cannot disappear (without an evidently sufficient cause) without a suspicion that it will be followed by something worse. It must follow, that this re-establishment of the regular asthmatic paroxysm was the sign of an improved state of the constitution." 252.

Dr. Lambe's mode of accounting for the annual recurrence of the paroxysm, is somewhat remarkable. He considers it a kind of exfoliation and regeneration of the membrane investing the bronchia and air-vessels of the lungs, a phænomenon observable on the external surfaces of the body: thus the epidermis peels off, and occasionally preserves its continuity; and the intestinal evacuations give incontestable evidence of the same fact, as every one must have observed membranes evacuated preserving the form of the intestine.

"The female head of Mr. Newton's family, to whose spirit, independence, and intelligence, the emancipation from the yoke of vulgar and destructive prejudices must be ascribed, enjoys an activity of mind and body rarely equalled in her sex. Our feeble and delicate country-women will, perhaps, be shocked, when they learn, that this lady, bred up in habits as delicate and luxurious as the most sensitive of themselves, has been enabled, during the course of this present year, to walk thirty miles in one day. She has a high colour, and is full of flesh. Such are the real mischiefs, and such the debility, which are the consequences of a vegetable regimen, when used by persons in good health, and of sound constitutions." 357.

Except some trifling ephemeral attacks, this family of children scarcely know what sickness is.

*Paralysis.* Mrs. O. ætat. 47, of a plethoric habit, suffered a paralytic attack of the left eye and cheek, in the spring of 1809. She had also frequent vertigo, and was under constant dread of fresh seizures. She had been cupped, bled, purged frequently and copiously, and put upon vegetable diet. By this plan her strength was impaired, while the disease remained the same. She was forced to wear a shade, in consequence of a morbid sensibility in the eye. She had great lowness of spirits ; the muscular strength entirely gone. She returned to her usual fare ; but was persuaded by our author to once more relinquish it, and confine herself to the vegeto-aqueous regimen. After this she remained debilitated for twelve months, with a wretched state of low spirits ; but she regained the power of closing the eye-lids, which had been lost. From this time, the amendment of the general health became more evident. From being pallid, she became rather florid, and was able to attend to her domestic affairs. The morbid sensibility of the eye was removed, and the shade was no longer necessary. The vertigo nearly disappeared, and the dejection of spirits vanished ; but the affection of the sensorium was only alleviated, frequent pains of the head recurred, for which she had often recourse to cupping.

"In this condition she has continued nearly ever since ; the general health rather improving than otherwise ; enjoying a state that is, comparatively, very comfortable.—This lady has neither lost flesh nor colour by abstaining from animal food : but her muscular strength is certainly diminished."

In a case of local disease, a medical gentleman adopted the vegeto-aqueous regimen recommended by our author, with relief of the affection, which was a small but painful tumour on the arm. Dr. L. has heard him assert, that, "for two years before he changed his diet, his spirits were so low, that he was unable to smile;" which lowness of spirits disappeared under the regime in question.

"It is no new observation (says Dr. L.) that vegetable diet has been useful in melancholic disorders. A case is given by Dr. Lobb, of a gouty pain of the stomach, with flatulence and melancholia, cured by vegetable diet. He has also been in the habit of illustrating the superiority of this regimen, by saying that the difference of comfort experienced between it and the common mode of life, is quite as great as what persons experience between the common mode of life and directly riotous living." 373.

*Scrofula.* Dr. Lambe very properly criticises several passages in the works of Dr. Beddoes, wherein vegetable diet is accused of being the grand cause of scrofula among the children of indigent parents. We believe that this opinion is daily losing ground, with the doctrines of debility and putrescency which gave stability to it. We often indeed see, at first, an increase of strength after the use of animal food; but this increased strength does not always continue, though the same diet be persevered in. "On the contrary, there is a sort of oscillation, the strength first rising, and then sinking again." We have often and often observed this. Dr. Lambe's opinions respecting the efficacy of distilled water and vegetable diet, in arresting the progress or mitigating the symptoms of carcinoma, are well known, and therefore we shall not dwell on them here.

A gentleman, under the signature "*Medicus*," gives Dr. L. an interesting account of the effects of the low regimen on himself, which we shall condense for our readers. When a boy, he took a disgust to animal food, and left it off, partly from hearing people talk of the health and longevity of the herbivorous nations. He believes he enjoyed as good health as before; and the increased pleasure which he began to take in literary and scientific employments at that time, inclines him to suspect, that a state of mind more friendly to mental enjoyments had probably been induced by the substitution of vegetable for animal food. About this time he fed, for a whole week or more, on raspberries, strawberries, and currants, during which period he never was better or stronger in his life. On the vegetable system he lost the dark incrustation of the teeth.

He left off this mode of living, however, in consequence of the inconvenience of using a diet different from those around him; but he resumed it again in 1811, while studying at Bartholomew's, where it was then the fashion among many of the pupils to live in the Brahmin manner. The regimen was now strict, and the change produced, at first, an augmentation of nervous sensibility, which was but temporary, as his health continued good, and he found himself more disposed for, and capable of laborious mental occupation than when feeding on a mixed diet. His muscular *strength* and *activity* were equally as great as before. He frequently walked twenty miles in a morning, and seldom felt fatigued.

"Whatever change (says Medicus) may be produced at first' a very similar state of health appears to return after the continuance of *any diet*, when eaten in moderation; at least, as far as temporary appearances indicate. How far a mixed diet lays secretly the foundation for future disorders, or may abridge the term of life, I am unable to say; but I am confident that people, in general, err considerably in the *quantity* of food they take, and the frequency of taking it, and the manner in which they stimulate their stomachs by spirituous and fermented liquors." 438.

In these sentiments we most unequivocally coincide with our anonymous writer.

To a person twenty-four years of age, harassed by a constant diarrhœa, for which animal food was prescribed, Medicus recommended the vegeto-aqueous regimen. He began at first by eating biscuits and other farinaceous substances, and by degrees habituated his stomach to vegetable diet. He grew healthy, and lost the diarrhœa. He then took to his old fare.

"I must say in conclusion (Medicus), that all I have observed of the good effects of vegetable, or any other diet, appears to me referrible to its power (arising either from idiosyncrasy, or some peculiar state of the patient's system) of tranquillizing stomachic and intestinal irritation; by this means, of insuring better digestion, and producing that tranquillity and healthy action of the chylo-poietic viscera, which is necessary to the cure of every disorder, whether general or local." 440.

*Hypochondriasis.* Under this head, Dr. L. presents us with a well written letter from JUSTINIAN MINOCH of Walworth; the substance of which, we shall give in our accustomed analytical brevity of language. Mr. M. made trial of the vegetable regime anterior to any knowledge of Dr. Lambe, and uninfluenced by any preconceived theory. Imperfect as was the trial at first, many unpleasant and intolerable sensations were alleviated. At our author's suggestion, he abandoned every thing animalized, and adopted a strict vegetable regimen, with distilled water; since which time, his health has sensibly increased, and is daily increasing. Felicity of mind, which had been despaired of, has been obtained, and ultimately there will be assured—"*quiete et pure et eleganter actæ ætatis placida ac lenis senectus.*"

At a very early period of life, and during the whole of his scholastic education, Mr. M's health was uncertain and precarious. The precise nature of his ailments, however, he cannot now recollect; between the age of thirteen and fourteen he suffered a severe attack of ICTERUS; for some years previous to which, distressing headaches, indigestion, and habitual constipation were experienced. To correct this "*bilious*" constitution, various remedies and means were tried without effect. At the age of fifteen he was removed from school, and for a series of years, laboriously and actively employed. During this period his health improved; but he relapsed into the hearty use of animal food and fermented liquors. After some years, his views and intentions in life having been changed, the scene of labour and activity was succeeded by sedentariness and study. Here his medical advisers recommended solids and liquids of a stimulating nature, to support the labour of the mind. The effects were soon visible: in a short time, he became wholly incapable of mental exertion; was harassed by giddiness and confusion of the head; disordered stomach, and irregularity of the bowels. His mind now became depressed, and disturbed by all the melancholy forebodings of exquisite hypochondriacism; experiencing, in short,

——— Mortis formidinem et iram,
Somnia, terrores magicos, miracula, sagas,
Nocturnos lemures portentaque Thessala!

Such was this gentleman's condition, sometimes better, sometimes worse, from 1802 till 1810; when, being at Edinburgh as a student of medicine, he was persuaded, by a fellow student, to adopt a vegetable regimen with milk, which, in six months, produced much alleviation of his sufferings. No very solid or substantial relief, however, was obtained till (in 1812) he substituted distilled water for milk. The change from that year has been great—all that had rendered existence irksome has been removed; his mind is tranquillized and calmed; his general health improved, and improving.

Mobilitate veget viresque acquirit eundo.

Through the past summer, he has frequently risen at four o'clock, to study, and gone to bed at ten, his sleep sound, refreshing, and free from horrid dreams. Not so when his food was flesh, and his drink fermented liquors: then the hours of sleep did not refresh him in mind, nor recruit him in body. He has observed that the lighter the food, and the more moderate in quantity, the more walking exercise he is equal to; moreover, the respiration is more equable, and slower in a given time, consequently the ability to *continue* exercise is increased.*

* "The lower or industrious classes, [among the Hindoos] on the other hand, who live almost exclusively on vegetables, certainly bear a striking resemblance to Pharoah's 'lean flesh kine.' But although they have not the physical strength of the European, they make up for this, in what

"Very different was it when I lived, not as at present; then there was wanted not only the inclination to exercise, but an ability to continue it: upon level ground, my respiration was frequent, hurried, laborious; now I can ascend a long and steep hill, walking very little slower than upon level ground. With respect to my bowels, they are now regular, requiring no medicine to excite them to action; whereas, when I lived otherwise than at present, they were torpid, and needed much stimulating." 448.

We recommend the perusal of this case to the serious consideration of the profession. Several letters follow, bearing testimony to the utility of vegetable diet and distilled water, in the cure or mitigation of human ailments; but we conceive it needless to quote them—sufficient has been adduced to bring conviction to the mind. Since Dr. Lambe's appointment to the General Dispensary, also, he has had opportunities, on a pretty large scale, of trying the principles and practice recommended, by the touchstone of experience, and a vast mass of satisfactory evidence is, accordingly, brought forward; for which, we refer those who may still be sceptical, to the volume itself.

### Conclusion.

It is not possible, Dr. L. thinks, to devise any other proof with regard to the agents which have the greatest influence on health, than that which has been given in the preceding pages. He has taken, as has been seen, examples of diseases acknowledged to be incurable, when they were presented in such a stage as to afford any rational prospect of relief, and he has given the results of experience. These observations, thus promiscuously taken, concur uniformly in corroborating the conclusions drawn from the diseases avowedly incurable by medicine. If, in these, the most sanguine hopes have not been entirely realized—if perfect cures have not been effected, nor the body restored to a complete state of health and integrity, still, it may be allowed, that what has been accomplished is neither trifling nor despicable. In cancerous diseases, particularly, to have relieved the excruciating torments of the malady;—to have prevented ulceration, with its

---

may be termed *bottom*; for it is well known, that a native will go through three times as much fatigue under a burning sky, as would kill an Englishman outright—witness the palankeen bearers, Coolies, Dandies, Hircarrahs, &c. &c.—*Johnson on Hot Climates*, page 436.

attendant miseries of loathsome, fœtid, and excoriating discharges;—to have preserved life, and that in such a degree of comfort as to enable the patient to enjoy society, and be equal to the common duties and occupations of the world; to have effected so much in cases where neither age, nor a completely broken down constitution present invincible obstacles to all amendment, is surely to have achieved much for suffering humanity; and amply compensates the proposer of this regimen for the anxiety and labour in which he has been involved—the obloquy of the ignorant, and the misrepresentations of the malevolent.

"It is neither pretended nor expected, that a morbid body can, by any art, be kept free from the attacks of disease. There seems to be in the body, as in vegetation, the seeds of future diseases, which continue latent and inactive for a length of time, they then germinate, increase, pass through their regular stages, and come to a termination. It is, however, beyond a doubt, that between that state of the body, in which there is merely a diseased disposition, and that consequent state in which disease becomes active, there is a long, though not a strictly definable interval. Thus the breath may begin to fail for three or four years before a person falls into a consumption. A change, therefore, takes place, certainly in the functions, and probably in the structure of the organs of respiration, long before the accession of confirmed cough and hectic fever. We see it more evidently in the cancer, in which there is pain, perhaps for a series of years, before there is any thickening of the parts." 488.

We have extended our review of Dr. Lambe's volume somewhat beyond its proportionate limits, because the subject is extremely interesting to society at large; and because the system of our author has been too often ridiculed as visionary, and his principles considered as founded in erroneous views of the animal economy. We are by no means disposed to treat with levity Dr. Lambe's theory or practice, convinced as we are, that, although he may carry his opinions beyond practicability, or even utility in some points, yet, that there is much solid truth in his reasonings, and that an adoption of his views would be of great benefit to mankind. But this, alas! is more to be wished than expected! Man is so much the slave of his passions and appetites, that were a prophet to rise from the tomb, and warn him against indulging them, he would not be listened to; or at least, his advice would be neglected. In the whole catalogue of human maladies, how many are brought on by deficiency in the quantity of food? Scarcely one! While, on the other hand, there is hardly a disease that is not ameliorated by diminishing the scale of our sustenance. Nature herself, in most instances, inculcates this important lesson. When any of the numerous tribe of acute and dangerous diseases approaches, the appetite is immediately withdrawn altogether; and, in a great proportion of chronic ailments, it is impaired. How have we improved this hint of Nature?—By not only turning a deaf ear to her voice, but by acting diametrically contrary to her salutary monitions. No sooner does the appetite fail, than the cook, the confectioner, and the chemist are up in arms to redress this grievous calamity; and caudles, condiments, and tonics, pave the way for bleeding, purging, and water-gruel!

> The first physicians by debauch were made;
> Excess began, and still sustains the trade.

In medical affairs, our attention is too often directed to effects instead of causes. Thus *debility* being a natural consequence of almost every derangement in the structure or function of the living machine, the great object of the patient, and too often of the physician, is to remove this *symptom*, very frequently at the expence of aggravating the original cause; to the removal of which, the debility is, in reality, favourable. Where lesion of an organ *suddenly* takes place, in a vigorous state of the system, we are forced to induce *debility* as quickly as possible; otherwise, the diseased organ will be in the greatest danger. Thus it is, that great strength and vigour of constitution are not only unfavourable to recovery from disease, but constantly predispose to it. How very seldom do we hear of valetudinarians, or people who are forced on a low regimen, dying in any sudden or mysterious manner? But does a week or a day pass, that we do not see blazoned forth in the public prints, or circulated in private, the accounts of men cut off in the prime of life and health, with scarcely any warning—generally, indeed, after a hearty dinner of animal food, some vigorous exertion, or a convivial supper! From what can these accidents arise, but too much blood [the consequence of too much food] bursting its natural boundaries, and overpowering some organ essential to life? Let the Brunonians and Carnivori ponder on these things!

